# Discussing the Debate: Leukocyte-Rich Platelet-Rich Plasma Versus Leukocyte-Poor Platelet-Rich Plasma

**DOI:** 10.7759/cureus.58381

**Published:** 2024-04-16

**Authors:** Sankalp Yadav

**Affiliations:** 1 Medicine, Shri Madan Lal Khurana Chest Clinic, New Delhi, IND

**Keywords:** leukocyte-rich prp, leukocyte-poor prp, leukocyte-rich vs. leukocyte-poor platelet-rich plasma, platelet-rich plasma (prp), leukocytes

## Abstract

This editorial touches on the persistent debate related to platelet-rich plasma (PRP) therapy. It focuses on the different approaches involving leukocyte-rich and leukocyte-poor PRP formulations of the PRP. It looks into the reasons behind these approaches, their potential effects on clinical practice, i.e., pros and cons, and the evidence supporting each method within the field of regenerative medicine. Examining these different views highlights the complexity of PRP therapy. It's important to proceed carefully, relying on evidence to enhance patient outcomes. Understanding the ongoing debates can help healthcare professionals make informed decisions. We need to consider various factors before implementing PRP therapy, ensuring it's done responsibly.

## Editorial

In regenerative medicine, platelet-rich plasma (PRP) therapy offers hope for tissue repair, and biologics like PRP have gained popularity in the last decade. Yet, challenges remain. Different PRP preparation methods yield varied formulations, and we lack data on the best formulations for specific therapies. The debate over PRP composition, leukocyte-rich versus leukocyte-poor, has raged on [1]. This editorial aims to explore these approaches, highlighting their unique traits, potential advantages, and clinical impacts.

Understanding leukocyte-rich PRP

Leukocyte-rich PRP is packed with white blood cells, platelets, and plasma. These cells are important parts of our immune system, as they swiftly respond against inflammation. In PRP, they're thought to turbocharge its immune-boosting abilities, fight off pesky microbes, and help tissues heal and remodel. Some folks believe adding more leukocytes makes PRP even better at treating injuries and inflammation [1,2]. But, there's a catch. A few studies hint that too many leukocytes might flare up inflammation due to an increase in the expression of inflammatory cytokines like tumor necrosis factor-alpha and interleukin-1, as well as catabolic cascades [1].

Exploring leukocyte-poor PRP

On the other side, leukocyte-poor PRP is all about lowering the number of white blood cells to prevent inflammation. This is achieved by giving it an extra spin in the centrifuge or using filters.

This method emphasizes the pivotal role of platelets in tissue regeneration, releasing essential growth factors including platelet-derived growth factor, insulin-like growth factor-1, transforming growth factor-β1, and vascular endothelial growth factor. Advocates of this approach posit that effective management of inflammation correlates with improved treatment results. This is especially true in certain situations where too much inflammation might slow down the healing process [3].

Clinical factors and evidence

In the field of medicine, leukocyte-rich and leukocyte-poor PRP are topics of discussion. Both of these PRPs have strong points to make. Numerous studies across orthopedics, sports medicine, dermatology, and dentistry have scrutinized the efficacy and safety of both PRP types. Some findings suggest that leukocyte-poor PRP can yield comparable or superior outcomes compared to leukocyte-rich PRP in specific contexts [1]. A small number of trials have found no benefit at all. Moreover, the decision between leukocyte-rich and leukocyte-poor PRP may be influenced by variables such as patient characteristics, the kind of injury or condition, and treatment procedures [1].

Future directions and clinical implications

It is essential to acknowledge that both leukocyte-rich and leukocyte-poor PRP have pros and cons. Clinicians should weigh individual patient needs, treatment goals, and available evidence when picking the right PRP type. It's crucial to recognize that even if we fix the leukocyte levels, factors like age, health status, gender, and ethnicity could affect PRP's effectiveness [4]. Moreover, more research, including randomized trials and comparative studies, is necessary to figure out the best PRP composition for different medical situations. Collaboration among researchers, clinicians, and regulators is key to establishing evidence-backed guidelines and standards for PRP therapy [5].

The ongoing debate surrounding leukocyte-rich versus leukocyte-poor PRP emphasizes the complex nature of PRP therapy and requires a meticulous approach for patient management. While both formulations promise tissue healing and regeneration, it's crucial to weigh clinical evidence alongside patient-specific variables and treatment objectives thoroughly. In the future, adopting a balanced and evidence-based strategy will be crucial for achieving the full therapeutic capabilities of PRP, thereby facilitating its seamless incorporation into clinical settings for patients' benefit.

In conclusion, the debate surrounding leukocyte-rich versus leukocyte-poor PRP shows the ongoing efforts for determining the optimized therapeutic strategies in regenerative medicine. By fostering dialogue, collaboration, and evidence-based practice, we can pass through this debate and pave the way for more effective and personalized PRP therapies in the future.
